# Characteristics and Associated Factors of Flat Irregular Pigment Epithelial Detachment With Choroidal Neovascularization in Chronic Central Serous Chorioretinopathy

**DOI:** 10.3389/fmed.2021.687023

**Published:** 2021-09-06

**Authors:** Yongyue Su, Xiongze Zhang, Yuhong Gan, Yuying Ji, Feng Wen

**Affiliations:** State Key Laboratory of Ophthalmology, Zhongshan Ophthalmic Center, Sun Yat-sen University, Guangzhou, China

**Keywords:** chronic central serous chorioretinopathy, flat irregular pigment epithelial detachment, type 1 choroidal neovascularization, RPE thickening, RPE aggregations

## Abstract

**Purpose:** Flat irregular pigment epithelial detachment (FIPED) in chronic central serous chorioretinopathy (CSC) is strongly associated with type 1 choroidal neovascularization (CNV). The present study aimed to describe the multimodal imaging characteristics of FIPED in patients with chronic CSC and investigate the factors associated with vascularized FIPED.

**Methods:** We included 55 chronic CSC eyes with vascularized FIPED (47 patients) and 55 chronic CSC eyes with avascular FIPED from age-matched patients (47 patients). None of the included eyes had a history of previous treatment with anti-vascular endothelial growth factor, photodynamic therapy, focal laser, or vitrectomy. The demographic and multimodal imaging data were reviewed. The location, angiography features, height and width, presence of retinal pigment epithelium (RPE) aggregations, RPE thickness, and choroid status of the FIPED area were compared between the groups.

**Results:** The mean age of the included chronic CSC patients was 54.3 ± 7.8 years (range: 33–72 years), and 85.1% were male. Vascularized FIPED eyes had a larger width (1,556.4 ± 731.6 vs. 931.1 ± 486.2 μm, *p* < 0.001), larger subfoveal RPE thickness (33.4 ± 15.3 vs. 26.3 ± 6.6 μm, *p* = 0.004), larger maximum RPE thickness of the FIPED area (46.3 ± 20.5 vs. 31.5 ± 8.3 μm, *p* < 0.001), and more RPE aggregations in the FIPED area (94.5 vs. 54.5%, *p* < 0.001) than avascular FIPED eyes. RPE aggregations in the FIPED area were an independent factor strongly associated with vascularized FIPED (OR = 7.922, 95% CI = 1.346–46.623, *p* = 0.022).

**Conclusion:** FIPED with a larger width and RPE thickening may suggest the presence of an underlying type 1 CNV. FIPED with RPE aggregations had an increased occurrence of neovascularization in chronic CSC.

## Introduction

Central serous chorioretinopathy (CSC), a chorioretinal disease that predominantly affects middle-aged men, is characterized by serous retinal detachment (SRD) that frequently involves the macular area and is associated with focal pigment epithelial detachment (PED) (1, 2). In chronic CSC, flat irregular PED (FIPED) is more frequently observed and presents as an irregular elevation of the retinal pigment epithelium (RPE) (3). FIPED is associated with type 1 choroidal neovascularization (CNV), which may lead to worse visual outcomes over long-term follow-up (4, 5).

The mechanisms underlying the development of type 1 CNV in FIPED (vascularized FIPED) remain unclear. Several factors have been shown to be risk factors for the formation of vascularized FIPED in CSC in prior studies, including chronic CSC, the female sex, choroidal vascular hyperpermeability (CVH), poor vision, and old age (6, 7); however, as one of the common manifestations of chronic CSC, the role of RPE disorders in CNV in chronic CSC has been overlooked. Some clinical and pathological studies of age-related macular degeneration (AMD) have pointed out that RPE-related changes, such as RPE migration and RPE thickening, could stimulate angiogenesis (8–10). The relationship between RPE disorders and vascularized FIPED remains to be elucidated. Moreover, age contributes to the irregularity of RPE cells and thinning of the choroid (11–13). In previous studies of CSC with CNV, age as a potential confounding factor may have led to bias in the assessment of RPE and the choroidal status in vascularized FIPED.

Previously, dye angiography has shown good accuracy in the detection of CNV; however, in regard to CNV in chronic CSC, the overlap of imaging features between type 1 CNV and CSC manifestations often challenges the detection of CNV (3). Optical coherence tomography (OCT) angiography (OCTA) is a non-invasive imaging modality that enables distinct depth-resolved 3D visualization of the choriocapillaris and retinal microvasculature (3, 14). Studies have shown that OCTA can detect CNV well in CSC and detect CNV more frequently than other imaging modalities (3, 14–16).

In the current study, we included chronic CSC eyes with vascularized FIPED and age-matched chronic CSC eyes with avascular FIPED to evaluate the FIPED features, RPE, and choroidal status on multimodal imaging and to further evaluate factors associated with vascularized FIPED.

## Materials and Methods

### Patients

This retrospective study was approved by the Institutional Review Board of Zhongshan Ophthalmic Center, and all research and data collection complied with the Declaration of Helsinki. Patients were informed of the risks of invasive examinations and signed informed consent forms.

Treatment-naïve chronic CSC eyes with vascularized FIPED and age-matched (within 5 years) chronic CSC eyes with avascular FIPED were included in this study from October 2018 to January 2021 at Zhongshan Ophthalmic Center. Chronic CSC was diagnosed as a neurosensory detachment caused by one or more sites of leakage at the level of the RPE at the first clinical visit and a record of persistent subretinal fluid (SRF) lasting 6 months or longer (1). FIPED was defined as an irregular elevation of the RPE and distinct visualization of Bruch's membrane on an OCT B-scan (3). The exclusion criteria were as follows: (1) drusen in the macular area; (2) type 2 and type 3 CNV; (3) high myopia of more than ±6 diopters (spherical equivalent); (4) other eye diseases, including glaucoma, polypoidal choroidal vasculopathy, retinal vein occlusion, or neurodegenerative disease; (5) history of previous treatment with anti-vascular endothelial growth factor (anti-VEGF), photodynamic therapy (PDT), focal laser, or vitrectomy; or (6) low-quality fundus images that could affect interpretation of the results.

### Image Acquisition and Analysis

Patients' clinical charts and multimodal imaging data were reviewed. All patients underwent a complete ophthalmic examination that included the following: best-corrected visual acuity (BCVA) examination, anterior segment examination, fundus biomicroscopy, and multimodal imaging examinations, including color fundus photography (FP) (FF450 plus; Carl Zeiss, Oberkochen, Germany), fundus autofluorescence (FAF), fundus fluorescein angiography (FFA), indocyanine green angiography (ICGA), OCT (Spectralis; Heidelberg Engineering, Heidelberg, Germany), and OCTA (RTVue 100; Optovue, Fremont, CA, USA). OCT scans were performed on single high-definition vertical and horizontal lines across the center of the fovea with a 30° area. Forty-nine B-scans that covered an area of 30° × 25° centered on the fovea were also obtained. The OCTA scanning area was captured in high-definition sections of 6 × 6 mm and 3 × 3 mm in size centered on the fovea.

Vascularized FIPED was determined by both en face OCTA and cross-sectional OCTA, which presented as a pathological flow in the outer retinal slab on en face OCTA and a hyperreflective, flat, irregular RPE elevation with a hyper-flow signal on cross-sectional OCTA (3). The automated segmentation line reference was manually fine-tuned if the automated segmentation was misaligned. OCTA images were evaluated at each successive slab to determine that the imaged abnormalities were real and not the result of projection artifacts. Two investigators (YS and XZ) independently evaluated the OCTA images to determine FIPED with or without CNV. Any disagreement was resolved by a senior retinal specialist (FW).

Multimodal imaging features of the FIPED area were qualitatively and quantitatively compared between vascularized FIPED and avascular FIPED. The FIPED location was classified as foveal involving or foveal sparing (17). The presence of fluorescence leakage on late-phase FFA, CVH, a neovascular choroidal network on early-phase ICGA, and intraretinal fluid (IRF) and SRF on OCT was assessed. The maximum height and width of the FIPED area were measured by the caliper function of OCT. The RPE and choroid status features of the FIPED area were further evaluated on OCT, including the subfoveal RPE thickness, the maximum RPE thickness of the FIPED area, the presence of RPE aggregations in the FIPED area (punctuate thickening of the RPE exceeding double the regular RPE height), the subfoveal choroidal thickness (SFCT), the thickness of Haller's layer under the FIPED area (the distance from the innermost point of the largest choroidal vessel to the inner border of the sclera), and the thickness of the choriocapillaris layer under the FIPED area (the distance from Bruch's membrane to the inner border of the large diameter choroidal vessels) (7, 18–21). The RPE and choroidal thickness were measured by the caliper function of OCT. All quantitative parameters were independently measured by two investigators (YS and YG), and the mean of the measurements was used for analysis.

### Statistical Analysis

Statistical analysis was performed using the Statistical Package for the Social Sciences for Windows ver. 25.0 (SPSS, Inc., Chicago, IL, USA). Continuous variables with a normal distribution are expressed as the mean ± standard deviation (SD) and were analyzed using independent *t*-tests. Categorical variables were compared using the chi-square test or Fisher's exact test, when appropriate. Multivariate logistic regression analysis was used to assess the factors associated with vascularized FIPED in chronic CSC, and the odds ratio (OR) and 95% confidence interval (CI) were calculated. *p*-values < 0.05 were considered significant.

## Results

### Patient and Ocular Characteristics

A total of 110 chronic CSC eyes with FIPED in 94 patients were included in this study. The demographic and clinical features of the included patients are summarized in Table 1. The patient age ranged from 33 to 72 years, with a mean (±SD) age of 54.3 ± 7.8 years. Most patients were male (80/94 patients, 85.1%). The mean BCVA of the included eyes was 0.39 ± 0.28 logMAR, and the mean duration from onset to last visit was 35.9 ± 33.7 months. The mean SFCT of the included eyes was 414.1 ± 118.2 μm. None of the 110 eyes had a history of previous treatment with anti-VEGF, PDT, focal laser, or vitrectomy. Fifty-five (50.0%) eyes of 47 patients had vascularized FIPED.

**Table 1:** Summary of the clinical and demographic features of included chronic CSC with FIPED.

	**Total or mean**
No. of patients (eyes)	94 (110)
Age (range), years	54.3 ± 7.8 (33–72)
Sex, male (%)	80 (85.1)
BCVA (range), logMAR	0.39 ± 0.28 (0.01–1.0)
Duration of onset (range), months	35.9 ± 33.7 (7–120)
SFCT (range), μm	414.1 ± 118.2 (163–857)
With vascularized FIPED, eyes (%)	55 (50.0)

*CSC, central serous chorioretinopathy; FIPED, flat irregular pigment epithelial detachment; BCVA, best-corrected visual acuity; SFCT, subfoveal choroidal thickness*.

### Characteristics of Eyes With Avascular and Vascularized Flat Irregular Pigment Epithelial Detachment

The mean age was 53.9 ± 7.8 and 54.6 ± 7.8 years (*p* = 0.683), and males accounted for 92% (38/47 patients) and 89.4% (42/47 patients) of patients (*p* = 0.386) in the avascular and vascularized FIPED groups, respectively. Clinical records and multimodal imaging features were compared between chronic eyes with avascular and vascularized FIPED (Table 2). Compared with the avascular FIPED group, the vascularized FIPED group showed a longer course of disease and poorer BCVA, but the differences were not statistically significant. In most cases, FIPED was located in the fovea in both the vascularized FIPED (44/55 eyes, 80.0%) and avascular FIPED (44/55 eyes, 80.0%) groups. On FFA, 72.7% (40/55 eyes) and 63.6% (35/55 eyes) of FIPED areas showed late-phase dye leakage in the avascular and vascularized FIPED groups, respectively. On ICGA, CVH was observed in almost all FIPED areas in both groups; the choroidal neovascular network was found in 36 (66.7%) eyes in the vascularized FIPED group on early-phase ICGA, and no neovascularization was detected in the avascular FIPED group. On OCT, the maximum width was significantly larger in the eyes with vascularized FIPED (1,556.4 ± 731.6 μm) than in the eyes with avascular FIPED (931.1 ± 486.2 μm; *p* < 0.001). The maximum height of FIPED was similar in the avascular FIPED group (65.1 ± 23.4 μm) and vascularized FIPED group (60.8 ± 23.5 μm). There was no significant difference in SRF or IRF above the FIPED areas between the groups.

**Table 2:** Comparison between avascular and vascularized FIPEDs in chronic CSC.

	**Avascular FIPED**	**Vascularized FIPED**	***p*-value**
	**(55 eyes)**	**(55 eyes)**	
Duration of onset, months	39.6 ± 38.6	45.8 ± 35.4	0.501
BCVA, logMAR	0.42 ± 0.28	0.36 ± 0.28	0.325
Location of FIPED, eyes (%)			1.000
Foveal involving	44 (80.0)	44 (80.0)	
Foveal sparing	11 (20.0)	11 (20.0)	
**Angiography features in FIPED area, eyes (%)**			
Fluorescence leakage on FFA	40 (72.7)	35 (63.6)	0.413
CVH	52 (94.5)	54 (98.2)	0.618
Neovascular choroidal network on ICGA	0 (0.0)	36 (66.7)	–
**OCT features in FIPED area**			
Maximum height of FIPED, μm	65.1 ± 23.4	60.8 ± 23.5	0.429
Maximum width of FIPED, μm	931.1 ± 486.2	1,556.4 ± 731.6	<0.001^*^
SRF above FIPED, eyes (%)	33 (60.0)	33 (60.0)	1.000
IRF above FIPED, eyes (%)	1 (1.8)	7 (12.7)	0.068

*FIPED, flat irregular pigment epithelial detachment; CSC, central serous chorioretinopathy; BCVA, best-corrected visual acuity; FFA, fundus fluorescein angiography; CVH, choroidal vascular hyperpermeability; ICGA, indocyanine green angiography; OCT, optical coherence tomography; SRF, subretinal fluid; IRF, intraretinal fluid*.

* *P < 0.05*.

The RPE and choroidal features were further compared between the avascular and vascularized FIPED groups (Table 3). The mean subfoveal RPE thickness in vascularized FIPED eyes (33.4 ± 15.3 μm) was significantly thicker than that in avascular FIPED eyes (26.3 ± 6.6 μm; *p* = 0.004). Regarding the FIPED area, the maximum RPE thickness (46.3 ± 20.5 vs. 31.5 ± 8.3 μm, *p* < 0.001) and presence of RPE aggregations (94.5 vs. 54.5%, *p* < 0.001) were significantly greater in vascularized FIPED areas than in avascular FIPED areas. The mean SFCT, thickness of the choriocapillaris layer, and thickness of Haller's layer beneath FIPED areas showed no significant differences between the groups. In the eyes with avascular and vascularized FIPED, the mean SFCT was 413.0 ± 112.3 and 415.0 ± 123.6 μm, respectively; the mean thickness of the choriocapillaris layer beneath FIPED areas was 53.0 ± 26.7 and 55.5 ± 38.4 μm, respectively; and the mean thickness of Haller's layer beneath FIPED areas was 307.8 ± 93.2 and 361.5 ± 95.3 μm, respectively.

**Table 3:** RPE and choroid status features of avascular and vascularized FIPEDs in chronic CSC.

	**Avascular FIPED**	**Vascularized FIPED**	***p*-value**
	**(55 eyes)**	**(32 eyes)**	
Subfoveal RPE thickness, μm	26.3 ± 6.6	33.4 ± 15.3	0.004^*^
Maximum RPE thickness of FIPED, μm	31.5 ± 8.3	46.3 ± 20.5	<0.001^*^
RPE aggregations of FIPED area, eyes (%)	30 (54.5)	52 (94.5)	<0.001^*^
SFCT, μm	413.0 ± 112.3	415.0 ± 123.6	0.938
Choriocapillaris layer thickness under FIPED, μm	53.0 ± 26.7	55.5 ± 38.4	0.742
Haller's layer thickness under FIPED, μm	307.8 ± 93.2	361.5 ± 95.3	0.050

*FIPED, flat irregular pigment epithelial detachment; CSC, central serous chorioretinopathy; RPE, retinal pigment epithelium; SFCT, subfoveal choroidal thickness*.

* *P < 0.05*.

**Figure 1:**
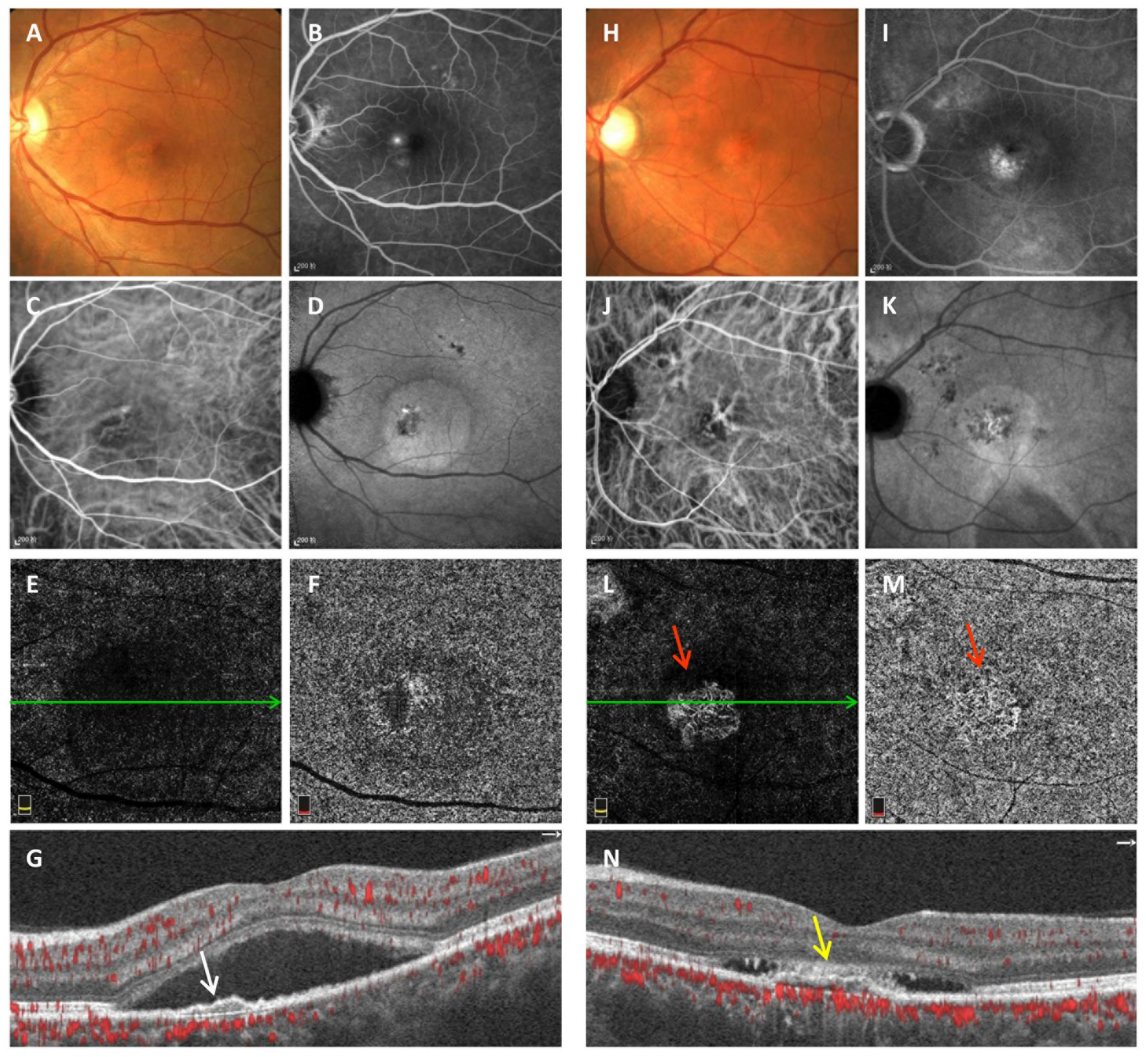
Multimodal imaging of avascular **(A–G)** and vascularized FIPED **(H–N)** in chronic CSC. Fundus photography **(A)** of avascular FIPED showed retinal neurosensory detachment in the macula. “Ink spots” dye leakage was observed on FFA **(B)**. ICGA showed dilation of choroidal vessels in the macular region in the early phase **(C)** and ovoid hyperfluorescence in the macula corresponding to retinal neurosensory detachment in the late phase **(D)**. No CNV signals were observed on ICGA **(C,D)**, in the outer retina layer **(E)** or choriocapillaris layer on OCTA **(F)**, or on cross-sectional OCTA [**(G)**, corresponding to the green line in **(E)**]. Cross-sectional OCTA showed that FIPED [white arrow in **(G)**] presented as an irregular elevation of the RPE. Fundus photography **(H)** of vascularized FIPED showed multifocal abnormal pigmentation. Dye leakage was observed on late-phase FFA **(I)**. Choroidal vessel dilation was shown in the macular region on early-phase ICGA **(J)**, but the choroidal neovascular network was not clearly shown. Both en face OCTA [red arrow in **(L,M)**] and cross-sectional OCTA [**(N)**, corresponding to the green line in **(L)**] showed CNV signals in vascularized FIPED [yellow arrow in **(N)**]. FIPED, flat irregular pigment epithelial detachment; CSC, central serous chorioretinopathy; FFA, fundus fluorescein angiography; ICGA, indocyanine green angiography; CNV, choroidal neovascularization; OCTA, optical coherence tomography angiography; RPE, retinal pigment epithelium.

Typical multimodal images of chronic CSC eyes with avascular and vascularized FIPED are shown in Figure 1. FIPED presented as an irregular elevation of the RPE allowing visualization of Bruch's membrane on cross-sectional OCTA (white arrow in G and yellow arrow in N). In chronic CSC with vascularized FIPED, FP (H), FFA (I), and ICGA (J, K) showed pigmentation in the FIPED area. The neovascularization within the FIPED area could be well-identified by the combination of en face OCTA (L) and cross-sectional OCTA (N) images.

### Multivariate Analysis of Factors Associated With Vascular Flat Irregular Pigment Epithelial Detachment

To determine the independent relevant factors of vascularized FIPED in chronic CSC, multiple logistic regression models were constructed. Single factors with *p* < 0.1 were included as variables in the multiple logistic regression analysis (Table 4). FIPED with RPE aggregations had an increased occurrence of neovascularization, by approximately 7.922-fold (OR = 7.922, 95% CI = 1.346–46.623, *p* = 0.022). In addition, the maximum width (OR = 1.002, 95% CI = 1.001–1.003, *p* = 0.005) and RPE thickness of FIPED areas (OR = 1.056, 95% CI = 1.005–1.109, *p* = 0.031) were significantly associated with the presence of vascularized FIPED.

**Table 4:** Multivariate analysis of factors associated with vascularized FIPED in chronic CSC.

**Variables**	**Univariate**		**Multivariate**	
	**OR (95% CI)**	***p*-value**	**OR (95% CI)**	***p*-value**
Maximum width of FIPED	1.002 (1.000–1.003)	<0.001^*^	1.002 (1.000–1.003)	0.005^*^
Subfoveal RPE thickness	1.082 (1.020–1.148)	0.009^*^	1.087 (0.999–1.182)	0.054
Maximum RPE thickness of FIPED	1.098 (1.044–1.156)	<0.001^*^	1.056 (1.005–1.109)	0.031^*^
RPE aggregations of FIPED area	14.444 (4.020–51.904)	<0.001^*^	7.922 (1.346–46.623)	0.022^*^
Haller's layer thickness under FIPED	1.006 (1.000–1.003)	0.056	1.002 (0.094–0.010)	0.595

*FIPED, flat irregular pigment epithelial detachment; CSC, central serous chorioretinopathy; RPE, retinal pigment epithelium*.

* *P < 0.05*.

## Discussion

In this study, 55 treatment-naïve chronic CSC eyes with vascularized FIPED and 55 treatment-naïve chronic CSC eyes with avascular FIPED from age-matched patients were included. In contrast to avascular FIPED eyes, the eyes with vascularized FIPED showed significantly more RPE aggregations and a significantly greater maximum RPE thickness in the FIPED area. The width of vascularized FIPED areas was also significantly larger than that of avascular FIPED areas. Our findings reveal that RPE thickening was associated with vascularized FIPED in chronic CSC.

**Figure 2:**
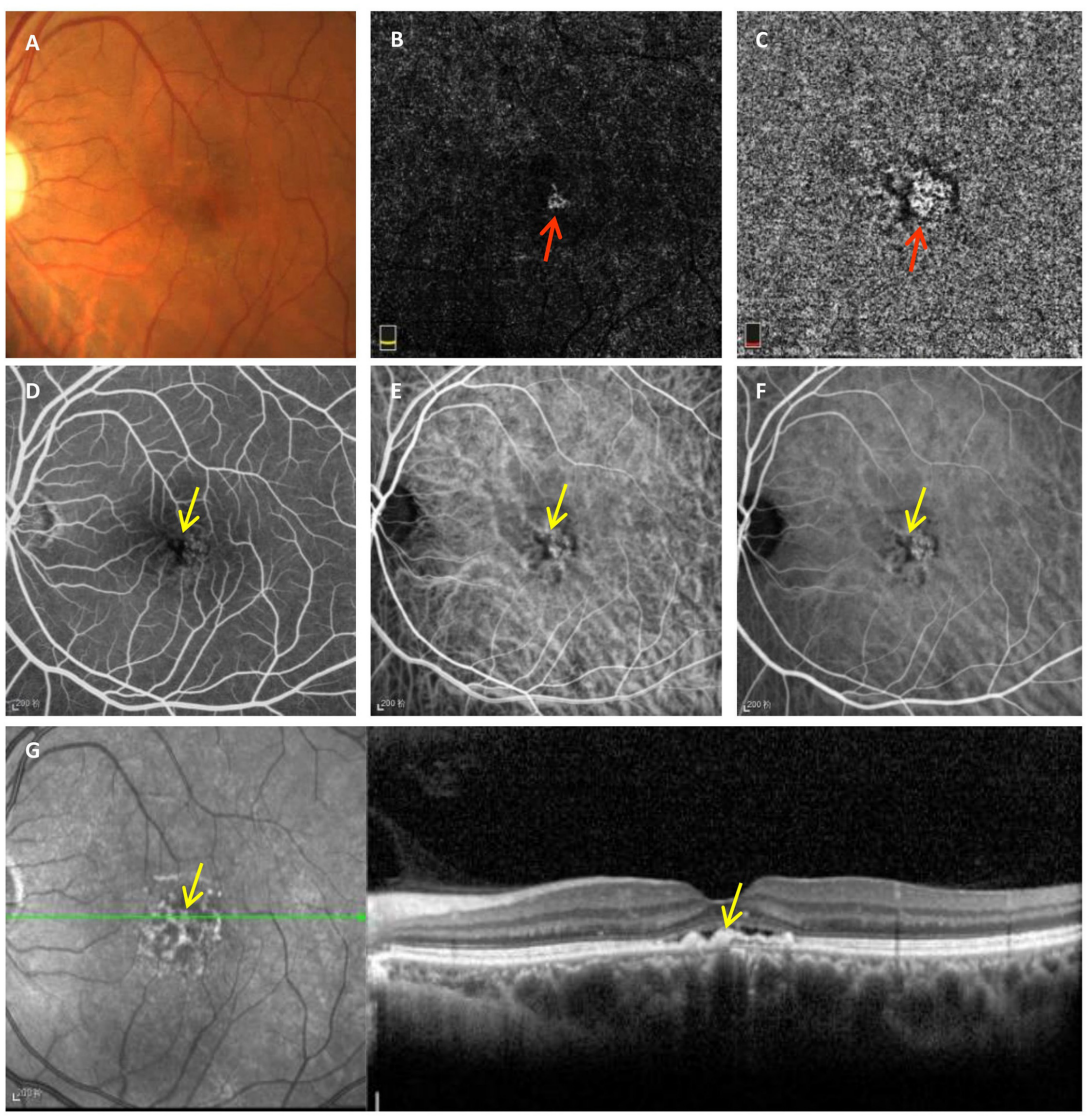
Typical manifestations of RPE thickening on multimodal imaging observed in chronic CSC with vascularized FIPED. Fundus photography **(A)** showed faint dark pigmented spots in the foveal area. En face OCTA of the outer retina **(B)** and choriocapillaris **(C)** showed CNV signals (red arrow). Early-phase FFA **(D)** showed blocked fluorescence (yellow arrow) in the fovea. Early-phase **(E)** and mid-phase ICGA **(F)** showed blocked fluorescence (yellow arrow) and choroidal vasodilation on the fovea. Hyperreflective dots on IR **(G)** and areas of RPE aggregations on OCT (yellow arrow) corresponded to regions with blocked fluorescence on FFA and ICGA, respectively. RPE, retinal pigment epithelium; CSC, central serous chorioretinopathy; FIPED, flat irregular pigment epithelial detachment; OCTA, optical coherence tomography angiography; CNV, choroidal neovascularization; FFA, fundus fluorescein angiography; ICGA, indocyanine green angiography; IR, infrared reflectance.

FIPED is defined as an irregular elevation of the RPE on OCT, and CNV may exist in the space between the RPE and Bruch's membrane (type 1 CNV) (3). Sarah et al. found that type 1 CNV is the most frequent subtype of CNV associated with chronic CSC and causes poor vision (4). Previous studies have revealed different pathological mechanisms between FIPED associated with and without CNV, although the mechanism is still unclear (3, 7). In this study, we found that RPE thickening was an independent factor strongly associated with vascularized FIPED in chronic CSC. RPE thickening manifested as irregular hyperreflective punctate or band-shaped areas of thickening of the RPE layer on OCT, which corresponded well with clinically observed hyperpigmentation, dark pigmented dots on fundus color photography, blocked fluorescence on FFA and/or ICGA, and hyperreflective foci on infrared reflectance (IR) (Figure 2) (8, 19).

In our chronic CSC eyes, vascularized FIPED showed significantly more RPE aggregations and a significantly greater maximum RPE thickness than avascular FIPED. Therefore, we speculate that the RPE may play an important role in the formation of vascularized FIPED in chronic CSC, and the possible pathogenic mechanism is as follows. In chronic CSC, the dilation of outer choroidal vessels produces an inward hydrostatic pressure that causes different degrees of RPE disorders, including RPE thickening (1, 19). RPE disorders could impede the passage of fluid and nutrients between the choroid and outer retina, resulting in relative ischemia (22, 23). Severe RPE thickening and the surrounding hypoxic conditions may result in excess VEGF secretion by the RPE, contributing to neovascularization (24). In addition, previous AMD studies have also shown evidence that RPE thickening occurs prior to CNV formation and is topographically and chronologically associated with CNV development (8). Moreover, in AMD studies, clinical hyperpigmentation corresponds to RPE hypertrophy on histopathology (25, 26).

Additionally, we found that vascularized FIPED was wider than avascular FIPED in chronic CSC, which is consistent with the findings of previous studies (3, 5, 7, 27). Furthermore, the SFCT, thickness of the choriocapillaris layer, and thickness of Haller's layer under FIPED areas were larger in the vascularized FIPED group than in the age-matched avascular FIPED group, but the differences were not significant. Pang et al. mentioned that thickening of the choroid may play an important role in type 1 CNV in CSC (28); however, Guo et al. and Kim et al. found a smaller SFCT in vascularized FIPED than in avascular FIPED (7, 29). We believe that these different results in terms of choroidal changes are mainly due to the age of the subjects. The choroidal thickness decreases with aging (30). In previous studies, age was found to be an independent risk factor for CNV secondary to chronic CSC, and the mean age of CNV patients was significantly greater than that of non-CNV patients (7, 27, 31). Therefore, CNV eyes with CSC often show a decreased choroidal thickness. In this study, we included patients with vascularized FIPED and age-matched patients with avascular FIPED to eliminate the effect of age on choroidal thickness; no significant difference in choroidal changes was found between the two groups, indicating that choroidal thickening in CSC may not be the only cause of CNV.

Recently, several studies have demonstrated that OCTA allows the detection of CNV in chronic CSC that is not visible with other imaging techniques due to overlapping of the imaging features on FFA and ICGA (3, 15, 16, 32). In this study, OCTA did not miss any cases of CNV that were detected by ICGA, but dye angiography was less effective than OCTA in CNV detection in chronic CSC, which is consistent with the findings of a study by Bousquet et al. (3). Our results also show that fluorescence leakage could occur in both vascularized and avascular FIPED. Both CNV and defects of the RPE in CSC could show poorly defined, late hyperfluorescence on FFA and ICGA, and the choroidal neovascular network of chronic CSC in early ICGA is difficult to identify because of the underlying choroidal vasodilation. Additionally, approximately one-third of vascularized FIPED areas were symptom-free and fluid-free, which could be the quiescent CNV (33). These findings often challenge the identification of CNV in chronic CSC by dye angiography (34).

Our research has potential limitations. First, as this was a hospital-based, retrospective study, there was selection bias. Second, further longitudinal observation and direct evidence of clinicopathological correlations are warranted to confirm that RPE thickening contributes to neovascularization in FIPED in chronic CSC to some extent. Despite these limitations, our study balanced the age of the participants and focused on the characteristics of FIPED. The results provide new insights regarding RPE thickening in the pathogenesis of vascularized FIPED in chronic CSC.

In conclusion, we found that vascularized FIPED areas in chronic CSC have a larger width, thicker RPE, and more RPE aggregations. Additionally, severe RPE thickening, such as RPE aggregations, is strongly associated with vascularized FIPED in chronic CSC.

## Data Availability Statement

The raw data supporting the conclusions of this article will be made available by the authors, without undue reservation.

## Ethics Statement

The studies involving human participants were reviewed and approved by The Institutional Review Board of Zhongshan Ophthalmic Center. The patients/participants provided their written informed consent to participate in this study.

## Author Contributions

YS, XZ, and FW contributed to the study conception and design. Material preparation, data collection, and analysis were performed by YS, XZ, YG, and YJ. The first draft of the manuscript was written by YS. All authors commented on previous versions of the manuscript, contributed to manuscript revision, read, and approved the submitted version.

## Conflict of Interest

The authors declare that the research was conducted in the absence of any commercial or financial relationships that could be construed as a potential conflict of interest.

## Publisher's Note

All claims expressed in this article are solely those of the authors and do not necessarily represent those of their affiliated organizations, or those of the publisher, the editors and the reviewers. Any product that may be evaluated in this article, or claim that may be made by its manufacturer, is not guaranteed or endorsed by the publisher.
